# Novel β-Lactamase *bla*_ARL_ in *Staphylococcus arlettae*

**DOI:** 10.1128/mSphere.00117-17

**Published:** 2017-05-03

**Authors:** Sabrina N. Andreis, Vincent Perreten, Sybille Schwendener

**Affiliations:** Institute of Veterinary Bacteriology, Vetsuisse Faculty, University of Bern, Bern, Switzerland; JMI Laboratories

**Keywords:** antibiotic resistance, beta-lactamases, coagulase-negative staphylococci, penicillinase

## Abstract

Whole-genome sequencing of penicillin-resistant *Staphylococcus arlettae* strain SAN1670 from bovine mastitis milk revealed a novel β-lactamase operon consisting of the β-lactamase-encoding gene *bla*_ARL_, the antirepressor-encoding gene *blaR1*_ARL_, and the repressor-encoding gene *blaI*_ARL_. The functionality of *bla*_ARL_ was demonstrated by gene expression in *Staphylococcus aureus*. The *bla*_ARL_ operon was chromosomally located in SAN1670 and present in 10 additional unrelated strains, suggesting intrinsic penicillin resistance in *S. arlettae*. Furthermore, a GenBank search revealed more unique potential β-lactamases in *Staphylococcus* species.

**IMPORTANCE** Penicillins are an important group of antibiotics used to treat various types of infections caused by Gram-positive bacteria. So far, the *blaZ* gene was the only known β-lactamase gene in staphylococci. However, other putative β-lactamases were identified, and one of them was shown to be a novel functional β-lactamase encoded by *bla*_ARL_ in *Staphylococcus arlettae*, further limiting treatment options.

## Abstract (precis)

Penicillins are an important group of antibiotics used to treat various types of infections caused by Gram-positive bacteria. So far, the *blaZ* gene was the only known β-lactamase gene in staphylococci. However, other putative β-lactamases were identified, and one of them was shown to be a novel functional β-lactamase encoded by *bla*_ARL_ in *Staphylococcus arlettae*, further limiting treatment options.

## OBSERVATION

*Staphylococcus arlettae* is a ubiquitous coagulase-negative staphylococcus first isolated from the skin and nares of poultry and goats, respectively (1). Later, it was also found in the environment of tobacco fermentation (Culture Collection, University of Göteborg [CCUG], Göteborg, Sweden), the skin of horses (2), and bovine teat skin (3). In some cases, it was associated with bovine mastitis (4). Today, the intramammary application of penicillin alone or in combination with other antibiotics is the mastitis treatment method most frequently used in dairy cows (5). However, penicillin can be hydrolyzed by β-lactamase-producing staphylococci that have acquired the *blaZ* gene, so far the only known β-lactamase gene in staphylococci (6). This gene is organized in an operon with the antirepressor-encoding gene *blaR1* and the repressor-encoding gene *blaI*. BlaR1 and BlaI form a regulatory two-component system responsible for inducible *bla*Z expression in the presence of β-lactam antibiotics (7, 8). The *blaZ* gene is widespread in several *Staphylococcus* species, including *Staphylococcus aureus* (6, 9), and has been found on different mobile genetic elements like transposon Tn*552* and conjugative plasmids (10–12).

In 2010, penicillinase-producing *S. arlettae* strain SAN1670 was isolated from a bovine mastitis milk sample at our institute in Switzerland. PCR failed to identify the *blaZ* gene, prompting us to determine the nature of this β-lactamase phenotype by whole-genome sequencing. This allowed us to identify a novel functional β-lactamase in *S. arlettae*. Searching for further *bla* homologs in the gene pool of *Staphylococcus* revealed several uncharacterized potential β-lactamase sequences.

## 

### Novel β-lactamase *bla*_ARL_ on the chromosome of *S. arlettae* SAN1670.

The whole-genome sequence of *S. arlettae* SAN1670 was obtained by using Illumina MiSeq technology and reagent kit v 2 (Illumina, Inc., San Diego, CA) at the Labormedizinisches Zentrum Risch, Liebefeld-Bern, Switzerland. Reads were assembled into contigs with Geneious version R9.1.5 (13). TBLASTn analysis (http://www.ncbi.nlm.nih.gov/blast/) of the contigs generated revealed a distantly related *blaZ* homolog on a 145-kb contig (GenBank accession number KY363215). This *blaZ* homolog was named *bla*_ARL_, where *bla* defines the gene and ARL is the enzyme, in accordance with the nomenclature used for other β-lactamases (14). The 849-bp *bla*_ARL_ gene encodes a 282-amino-acid protein containing the consensus pattern for the β-lactamase class A active site (PS00146) defined in the Prosite database (15). The active-site serine present in all class A, C, and D β-lactamases was identified at position 63 of the ARL enzyme. The *bla*_ARL_ gene was preceded by two regulatory genes, *blaI*_ARL_ and *blaR1*_ARL_, transcribed in the opposite direction, forming a β-lactamase operon similar to *blaI-blaR1-blaZ*. This operon had 55% overall nucleotide sequence identity with Tn*552* (GenBank accession number X52734) (11) and is expected to be responsible for inducible *bla*_ARL_ expression in *S. arlettae* SAN1670. Analysis of a 50-kb region on each side of the *bla*_ARL_ gene identified genes belonging to the core genome of staphylococci such as *xprI*, *pbuX*, *guaA*, and *guaB*, which are involved in purine metabolism, as well as *rpsR*, *rpsF*, and *ssb*, which encode ribosomal proteins and a single-strand DNA-binding protein. The absence of transposases or recombinases within this region indicates that *bla*_ARL_ is stably integrated into the chromosome.

### Identification of *bla* homologs in staphylococci.

**FIG 1:**
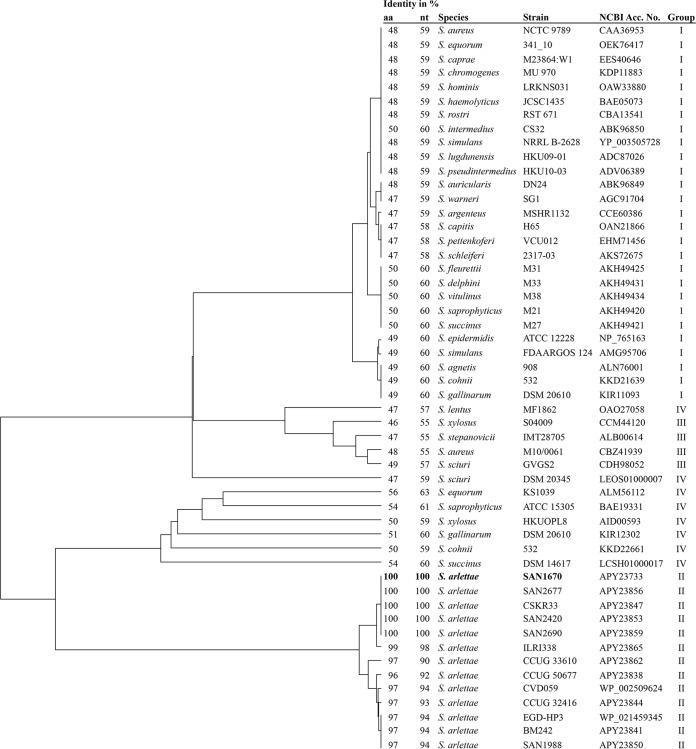
Phylogenetic tree of β-lactamases encoded by staphylococci. Evolutionary analysis was performed for amino acid sequences by the unweighted pair group method using average linkages in MEGA7. Evolutionary distances were computed by the Poisson correction method and were measured as the number of amino acid substitutions per site. The percentages of amino acid and nucleotide sequence identity between *bla*_ARL_ and other β-lactamases were determined by sequence alignment with Clustal Omega (http://www.ebi.ac.uk/Tools/msa/clustalo/). Roman numerals indicate β-lactamase groups as follows: I, *bla*Z; II, *bla*_ARL_; III, β-lactamases of the class E *mec* gene complex; IV, group of diverse uncharacterized β-lactamases.

A search for ARL enzyme homology in all of the available staphylococcal sequences in the NCBI GenBank database showed that the *bla*_ARL_ gene was also present in shotgun genomes of *S. arlettae* strains CVD059 (GenBank accession number ALWK01000016) (16) and EGD-HP3 (GenBank accession number AVOQ01000023). These *bla*_ARL_ genes were 99.5% identical and had 94% nucleotide sequence identity and 97% amino acid sequence identity with *bla*_ARL_ of SAN1670. Alignment of *bla*_ARL_ with *blaZ* of *S. aureus* NCTC 9789 (GenBank accession number X52734) (11) resulted in only 59% nucleotide sequence identity between the genes and 48% amino acid sequence identity between the β-lactamases ARL and PC1 encoded by *blaZ*. The PC1 enzyme is widespread in staphylococci and was identified in 27 different species (Fig. 1). Additional putative β-lactamases containing the class A consensus pattern (PS00146) were also detected. Four of these β-lactamases were found in the class E *mec* gene complex and clustered into a group with 67 to 71% amino acid sequence identity with PC1 and 46 to 49% amino acid sequence identity with ARL (Fig. 1). The other eight uncharacterized β-lactamases were unrelated and had 47 to 67% amino acid sequence identity with PC1 and 47 to 56% amino acid sequence identity with ARL (Fig. 1). These putative β-lactamases were unique to the species they belonged to, and none of them were preceded by the regulatory genes *blaI* and *blaR1*, such as in *blaZ* and *bla*_ARL_ operon.

### Expression of *bla*_ARL_ in *S. aureus*.

To prove the functionality of the novel β-lactamase of *S. arlettae*, the *bla*_ARL_ gene was cloned with and without the regulator genes *blaI*_ARL_ and *blaR1*_ARL_ from SAN1670 and expressed in *S. aureus* RN4220. The entire *blaI*_ARL_-*blaR1*_ARL_-*bla*_ARL_ operon was amplified with primers blaR1_M1670-XhoI-F and bla_M1670-PstI-R (see Table S1 in the supplemental material for the primers and PCR conditions used). The resulting fragment was cloned into the XhoI and PstI restriction sites of the *S. aureus-Escherichia coli* shuttle vector pTSSCm (17) to generate plasmid pSAN01. The *bla*_ARL_ gene alone was amplified with primers bla_M1670-NdeI-F and bla_M1670-SpeI-R (see Table S1) and inserted downstream of the type 1 capsule gene 1A promoter (P_*cap*_) of pBUS1-P_*cap*_-HC (17) to generate plasmid pSAN02. Plasmids pSAN01 and pSAN02 were transformed into *E. coli* DH5α and selected for tetracycline resistance (10 µg/ml) encoded on the vectors. Sanger sequencing confirmed the correct *bla*_ARL_ operon sequence in pSAN01; therefore, the plasmid was electroporated into RN4220 (18). However, nonsense mutations were observed at the 5′ end of the *bla*_ARL_ gene in all of the pSAN02 plasmids sequenced, indicating that constitutive β-lactamase expression could be deleterious to *E. coli*. To reverse the mutation in *bla*_ARL_ from pSAN02, QuikChange site-directed mutagenesis was performed directly in *S. aureus* RN4220. A missing thymidine (T) in the T stretch at gene positions 10 to 15 in a faulty plasmid was introduced by PCR (Phusion Hot Start II High-Fidelity DNA Polymerase; Thermo Fisher Scientific, Waltham, MA) with overlapping primers mut_M1670-F (5′-GGTTTATCAT**ATG**AAAAAG*TTTTTT*ACTATCTTTGTCTTACTCTG) and mut_M1670-R (5′-CTTTTT**CAT**ATGATAAACCTCCTATTTTCCTTTCTTGTTTTC) (the T stretch is italic, and the start codon of *bla*_ARL_ is bold) (19). The reaction product was treated with the DpnI restriction enzyme and directly electroporated into RN4220 cells to obtain plasmid pSAN02mut. Sequencing of the mutagenized plasmid in RN4220 clones confirmed the correct sequence of *bla*_ARL_. Furthermore, pSAN02mut isolated from RN4220 could not be transformed into *E. coli*, confirming that the constitutive expression of *bla*_ARL_ from P_*cap*_ is not compatible with *E. coli*.

10.1128/mSphere.00117-17.1 TABLE S1  Primers and PCR conditions used in this study. Download TABLE S1, PDF file, 0.3 MB. Copyright © 2017 Andreis et al. 2017 Andreis et al. This content is distributed under the terms of the Creative Commons Attribution 4.0 International license.

The production of a functional β-lactamase by *S. aureus* RN4220 containing pSAN01 and pSAN02mut was demonstrated by a positive nitrocefin test on BBL DrySlide nitrocefin (Becton, Dickinson and Company, Franklin Lakes, NJ) and by increased resistance to penicillin (Table 1) but not to other β-lactams, including ceftriaxone, cefaclor, cefepime, cefixime, cefuroxime, ertapenem, cefepime, cefotaxime, imipenem, ceftazidime, and temocillin. MICs were determined by microdilution in cation-adjusted BBL Mueller-Hinton II Broth (Becton, Dickinson and Company) with EUST, HPB1, and EUVSEC2 Sensititre Plates (Thermo Fisher Scientific) in accordance with CLSI guidelines (20).

**TABLE 1:** *Staphylococcus* strain characteristics and origins and MICs of β-lactam antibiotics

Strain/plasmid	Origin and characteristics	Reference or source	MIC (µg/ml)				Nitrocefin test result
			Penicillin	Ampicillin	Cefoxitin	Meropenem	
*S. aureus*							
RN4220	Plasmid-free recipient	25	≤0.125	≤0.12	2	0.06	Negative
RN4220/pBUS1-P_*cap*_-HC	RN4220 containing expression vector pBUS1-P_*cap*_-HC	17	≤0.125	≤0.12	2	0.06	Negative
RN4220/pTSSCm	RN4220 containing cloning vector pTSSCm	17	≤0.125	≤0.12	2	0.06	Negative
RN4220/pSAN01	RN4220 harboring pTSSCm with *blaI*_ARL_-*blaR1*_ARL_-*bla*_ARL_ operon	This study	0.25	≤0.12	2	0.06	Positive
RN4220/pSAN02mut	RN4220 harboring pBUS1-P_*cap*_-HC with *bla*_ARL_ gene under control of P_*cap*_ promoter	This study	2	0.5	4	0.12	Positive
*S. arlettae*							
SAN1670	Bovine mastitis milk, Switzerland, 2010	This study	0.5	0.5	4	0.5	Positive
SAN2677	Bovine mastitis milk, Switzerland, 2015	This study	0.25	0.5	4	0.25	Positive
SAN2690	Bovine mastitis milk, Switzerland, 2015	This study	0.25	0.5	4	0.25	Positive
SAN1988	Bovine mastitis milk, Switzerland, 2016	This study	0.5	0.25	2	0.25	Positive
SAN2420	Bovine mastitis milk, Switzerland, 2016	This study	0.5	0.5	2	0.5	Negative
BM242	Bovine mastitis milk, Switzerland, 2016	This study/Agroscope	0.25	0.5	4	0.5	Positive
CSKR33	Equine skin, Switzerland, 2004	2	0.5	1	2	0.25	Positive
CCUG 33610	Tobacco fermentation process, Sweden, 1994	CCUG, 1994	0.25	0.25	4	0.25	Positive
CCUG 50677	Tobacco, Sweden, 2005	CCUG, 2005	0.25	0.5	2	0.25	Positive
CCUG 32416 ^T^	Poultry skin, Belgium, 1984	1	0.25	0.25	2	0.25	Positive
ILRI338	Camel nasal cavity, Kenya, 2014	This study/ILRI	0.25	0.25	4	0.25	Positive

The MICs of both penicillin and ampicillin were higher for RN4220/pSAN02mut expressing *bla*_ARL_ constitutively than for RN4220/pSAN01 containing *bla*_ARL_ regulated by *blaI*_ARL_ and *blaR1*_ARL_ (Table 1). Higher MICs of the cephalosporin cefoxitin and the carbapenem meropenem, with a 2-fold increase, were also observed with pSAN02mut. This is likely to be a side effect of overproduction of ARL, a protein that can bind β-lactams. It is unlikely that ARL can hydrolyze these β-lactam rings since class A β-lactamases like PC1 are primarily penicillinases and are not expected to have any cephalosporinase or carbapenemase activity (21). Absence of carbapenemase activity was confirmed with the Blue-Carba test (22).

### Distribution of *bla*_ARL_ in *S. arlettae*.

Ten additional *S. arlettae* strains from different origins were tested for β-lactam resistance (Table 1). All displayed decreased susceptibility to penicillin with a MIC above the CLSI resistance breakpoints (20). Production of β-lactamase by the nitrocefin slide method was also observed in all of the strains except SAN2420, which was negative in this test. All strains were positive for *bla*_ARL_ by PCR with primers blaARL-F (5′-CTATCTTTGTCTTACTCTGTGT) and blaARL-R (5′-GCMTGACGTGCTGCTTGTGC) (see Table S1). Analysis of the *bla*_ARL_ region by PCR and Sanger sequencing revealed an intact *blaI*_ARL_-*blaR1*_ARL_-*bla*_ARL_ operon. The operon was located between open reading frames encoding a MaoC-like domain-containing protein and a peptide ABC transporter permease, the same as in the sequenced strains SAN1670, CVD059, and EGD-HP3 (see Table S1). The *blaI*_ARL_-*blaR1*_ARL_-*bla*_ARL_ operon sequences of the 10 *S. arlettae* strains have 88 to 100% nucleotide sequence identity with that of SAN1670.

The universal presence of *bla*_ARL_ in all of the tested *S. arlettae* strains from different sources suggests intrinsic penicillin resistance in this species. The *blaI*_ARL_-*blaR1*_ARL_-*bla*_ARL_ operon seems to be a stable part of the core genome and not to be associated with any recombinase. However, the location between *guaA* and *rpsR*, integration hot spots for genomic islands (23, 24), suggests a potential for *bla*_ARL_ mobilization. In addition, diverse proteins containing typical β-lactamase motifs appear to be present in many different *Staphylococcus* species. They lack the antirepressor *blaR1* and repressor *blaI* genes, and their role in β-lactam resistance is unclear. Our data propose a broader genetic analysis of penicillin-resistant staphylococci that do not contain *blaZ*. They also show that the presence of a functional β-lactamase in *S. arlettae* is presumable and jeopardizing penicillin treatment. The identification of the pathogen, as well as antimicrobial susceptibility testing, is therefore necessary for correct and effective therapy.

### Accession number(s).

The sequence of the *bla*_ARL_-containing contig of *S. arlettae* SAN1670 has been deposited in the GenBank database under accession number KY363215. The sequence of the *blaI*_ARL_-*blaR1*_ARL_-*bla*_ARL_ operon of *S. arlettae* strain ILRI338 has been deposited under accession number KY464892, and those of strains CCUG 50677, BM242, CCUG 32416, CSKR33, SAN1988, SAN2420, SAN2677, SAN2690, and CCUG 33610 have been deposited under accession numbers KY363206 to KY363214, respectively.
