# Supplementary material for: Novel β-Lactamase blaARL in Staphylococcus arlettae
Source: mSphere. 2017 May 3;2(3):e00117-17. doi: 10.1128/mSphere.00117-17 (PMC5415633; doi:10.1128/mSphere.00117-17)
Supplement: TABLE S1 [file sph003172281st1.pdf]

# Supplemental material

## Table S1. PCRs and conditions

| Primer Name | Primer Sequence 5'-3'[a] | Polymerase | Annealing temperature | Target |
|---|---|---|---|---|
| blaR1_M1670-XhoI-F | TGAATCTCGAGCTAAAACATTTATAGTTACGCATCAG | *Pfu* DNA Polymerase (Promega Corporation, Madison, WI) | Cycle 1-4: 53°C<br>Cycle 5-26: 59°C | *blaI*$_{ARL}$-*blaR1*$_{ARL}$-*bla*$_{ARL}$ operon |
| bla_M1670-PstI-R | TCTCTCTGCAGCTCTAAGTGCTGTTGGTT | | | |
| bla_M1670-NdeI-F | TCTATCATATGAAAAAGTTTTTTACTATCTTTGTCTTACTC | *Pfu* DNA Polymerase | Cycle 1-4: 53°C<br>Cycle 5-26: 59°C | *bla*$_{ARL}$ |
| bla_M1670-SpeI-R | TATATACTAGTCAATCGAGCTGATGTATTGCATG | | | |
| mut_M1670-F | GGTTTATCAT**ATG**AAAAAG*TTTTTT*ACTATCTTTGTCTTACTCTG | Phusion™ Hot Start II High-Fidelity DNA Polymerase (Thermo Fisher Scientific, Waltham, MA) | 65°C | *bla*$_{ARL}$ |
| mut_M1670-R | CTTTTT**CAT**ATGATAAACCTCCTATTTTCCTTTCTTGTTTTC | | | |
| ARL_ABCtransp-F | GCCATTGGCTAAACCCGATA | FIREPol® DNA Polymerase (Solis Byodine, Tartu, Estonia) | 58°C | *ABC transporter permease – blaI*$_{ARL}$ |
| blaIARL-R | GATGGCGTTTATCAGTACACACC | | | |
| blaIARL33610-R[b] | GCTGGCGTTTATCAATATACGCC | | | |
| ARL_MaoC-R | GCCATTGGCTAAACCCGATA | FIREPol® DNA Polymerase | 60°C | *bla*$_{ARL}$ – *maoC* |
| blaARL-F3 | GTWGCACAAGCAGCACGTCA | | | |
| blaARL-F | CTATCTTTGTCTTACTCTGTGT | FIREPol® DNA Polymerase | 55°C | *bla*$_{ARL}$ |
| blaARL-R | GCMTGACGTGCTGCTTGTGC | | | |

[a] Restriction sites used for cloning are underlined, start codon of *bla*$_{ARL}$ is in bold

[b] Different primer used for strain CCUG 33610 due to nucleotide diversity in *blaI*$_{ARL}$
